# dsAMP and dsAMPGAN: Deep Learning Networks for Antimicrobial Peptides Recognition and Generation

**DOI:** 10.3390/antibiotics13100948

**Published:** 2024-10-09

**Authors:** Min Zhao, Yu Zhang, Maolin Wang, Luyan Z. Ma

**Affiliations:** 1 State Key Laboratory of Microbial Resources, Institute of Microbiology, Chinese Academy of Sciences, Beijing 100101, China; zhaomin21a@mails.ucas.ac.cn (M.Z.); zhangyu_0824@outlook.com (Y.Z.); 2 University of Chinese Academy of Sciences, Beijing 100049, China; 3 Department of Bioscience and Biotechnology, Graduate School of Bioresource and Bioenvironmental Sciences, Kyushu University, 744 Motooka, Nishi-ku, Fukuoka 819-0395, Japan; 4 CAAC Key Laboratory of General Aviation Operation, Civil Aviation Management Institute of China, Beijing 100102, China

**Keywords:** antimicrobial peptides, deep learning, transformer, bioinformatics, sequence analysis

## Abstract

Antibiotic resistance is a growing public health challenge. Antimicrobial peptides (AMPs) effectively target microorganisms through non-specific mechanisms, limiting their ability to develop resistance. Therefore, the prediction and design of new AMPs is crucial. Recently, deep learning has spurred interest in computational approaches to peptide drug discovery. This study presents a novel deep learning framework for AMP classification, function prediction, and generation. We developed discoverAMP (dsAMP), a robust AMP predictor using CNN Attention BiLSTM and transfer learning, which outperforms existing classifiers. In addition, dsAMPGAN, a Generative Adversarial Network (GAN)-based model, generates new AMP candidates. Our results demonstrate the superior performance of dsAMP in terms of sensitivity, specificity, Matthew correlation coefficient, accuracy, precision, F1 score, and area under the ROC curve, achieving >95% classification accuracy with transfer learning on a small dataset. Furthermore, dsAMPGAN successfully synthesizes AMPs similar to natural ones, as confirmed by comparisons of physical and chemical properties. This model serves as a reliable tool for the identification of novel AMPs in clinical settings and supports the development of AMPs to effectively combat antibiotic resistance.

## 1. Introduction

Antimicrobial peptides (AMPs) are an integral part of the innate immune system and offer a promising alternative to traditional antibiotics [1,2]. Known for their rapid bactericidal action, low toxicity, and broad spectrum of activity, AMPs have significant therapeutic potential [3]. Their structure, with hydrophobic and amphiphilic residues, enables effective interaction with microbial membranes, resulting in cell disruption and death. AMPs also enhance therapeutic efficacy by synergizing with antibiotics and the host immune response [4]. With the growing threat of antimicrobial resistance, there is an urgent need to harness AMPs to combat this global health crisis. With their diverse activities against various pathogens, including cancer cells, AMPs are central to biomedical research [5]. Therefore, the identification and understanding of novel AMPs and their properties are critical to the development of potent antimicrobial agents and the advancement of therapeutic interventions.

In recent years, bioinformatics has made significant progress in the rational design of antimicrobial peptides (AMPs) using predictive methods based on sequence data [6]. Various algorithms, in particular machine learning models such as support vector machines (SVM) [7], random forests (RF) [8], and discriminant analysis (DA) [6], are commonly used for AMP prediction. Despite their effectiveness, these methods often struggle with feature extraction from protein sequences, which affects model performance. To overcome these challenges, deep learning techniques have emerged that provide automatic feature extraction from raw data. Deep learning models such as convolutional neural networks (CNNs) [9] and long short-term memory networks (LSTMs) [10] excel at capturing complex patterns within protein sequences, thereby improving AMP prediction accuracy. In addition, comprehensive AMP databases such as APD3 [11], AMPFun [4], dbAMP [12], DRAMP [13], and LAMP [14] have been established. The integration of machine learning classifiers and deep learning predictors has streamlined the identification and classification of potential AMP candidates. These computational approaches employ different feature selection strategies and algorithms to distinguish AMPs from non-AMPs, improving the efficiency of screening and prediction. As computational methods evolve and AMP databases grow, accurate identification and classification of AMPs by computational approaches will become increasingly important for advancing research to combat antimicrobial resistance [15].

The development of new antimicrobial peptides (AMPs) is crucial, but their design faces challenges due to the lack of predefined structural patterns and specific physicochemical property preferences [16]. Recent advances in deep learning have transformed the approach to data-driven biological problems, including the rapid discovery of new AMPs. For example, Dean and Walper used a variational autoencoder (VAE) to generate novel AMPs [17]. Natural language models adapted to proteins, such as those using LSTM and attention mechanisms, have also been instrumental in capturing AMP features [18]. In addition, Generative Adversarial Networks (GANs) [19] have made significant contributions to biological research by improving AMP design and genomics. Notable applications include PepGAN [20], which generates AMPs, and WGAN-based architectures for designing DNA sequences with desired properties [21].

Machine learning (ML) and deep learning techniques have gained significant traction in biological sequence analysis. These methods involve converting protein sequences into machine-readable formats and encoding features for digital mapping using signal processing approaches [17,22]. Various encoding strategies have been explored, such as the PC6 protein encoding method [23] and pseudo amino acid composition (PseAAC) [24]. In addition, the influence of pre-trained models from natural language processing, such as Protrans [25] and word2vec [26], is expanding into protein sequence analysis.

Building on previous research, we present dsAMP, an advanced AMP classification model that significantly enhances AMP sequence prediction. dsAMP combines the strengths of previous protein classification models, leverages a protein pre-training model on large datasets, and integrates advanced components such as CNNs, attention mechanisms, and bi-directional long short-term memory (BiLSTM). Our model demonstrates substantial improvements in accuracy, sensitivity, and specificity for both AMP sequence and functional recognition. Additionally, dsAMP effectively predicts AMPs through transfer learning on diverse bacterial datasets, even with limited data, further improving the wide application of dsAMP.

**Figure 1:**
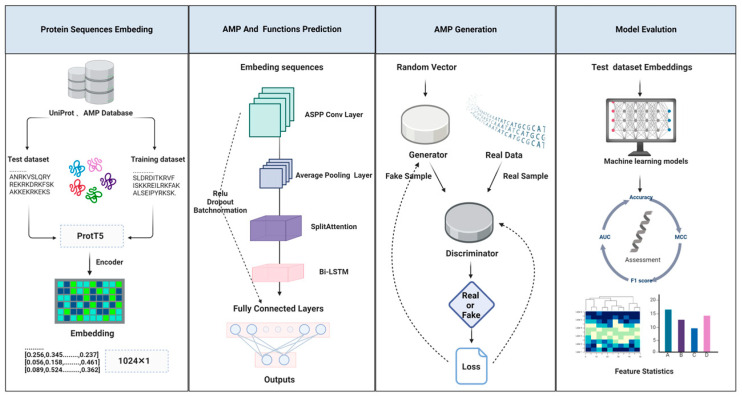
Overall architecture of dsAMP and dsAMPGAN.

To further advance AMP development, we introduce dsAMPGAN, a novel GAN-based AMP generation model. dsAMPGAN excels in generating AMPs with properties akin to the original peptides, including hydrophobicity, aromaticity, and net charge. As illustrated in Figure 1, our comprehensive workflow highlights the unique contributions and advancements of dsAMP and dsAMPGAN. The combination of these two models can accelerate the discovery of novel AMPs and advance the development of antimicrobial drugs, especially in the face of the growing threat of drug resistance. This approach not only improves the accuracy of AMP design but also expands the application potential of AMPs, which is important in the context of antimicrobial resistance.

## 2. Results

### 2.1. Sequence Analysis of the Datasets

**Figure 2:**
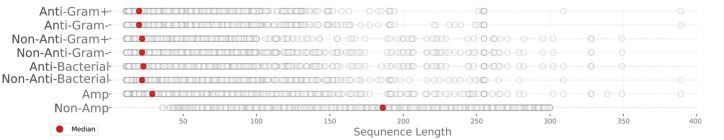
Percentage distribution of sequence length ranges based on datasets. Gray circles depict various sequences within the dataset, while red circles denote the median sequence length.

**Figure 3:**
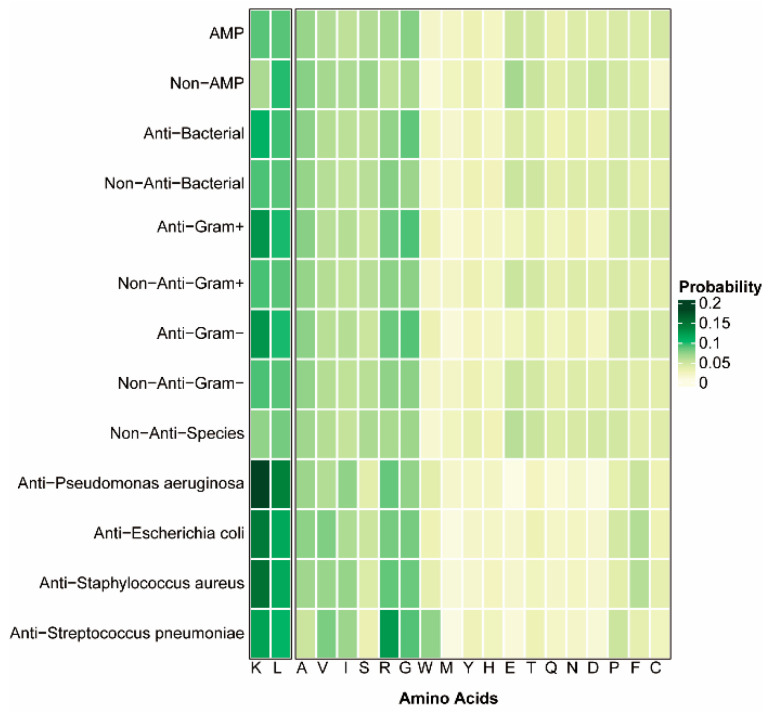
Amino acid distributions of datasets.

Sequence analysis of the datasets provided valuable information for understanding their composition. Figure 2 and Figure 3 show the amino acid sequence length and distribution of the constructed positive and negative datasets. Apart from the non-AMP sequences, there were no substantial length variations observed between positive and negative AMP samples across diverse functional datasets, with the majority exhibiting lengths below 50. In addition, a unique pattern was observed when looking at the abundance of different amino acids. Leucine (L), lysine (K), and arginine (R) residues were significantly more abundant in the sequences of the positive samples compared to the negative samples. Notably, these amino acids were either positively charged or hydrophobic, a feature consistent with the typical composition of previously studied AMPs [3]. This arrangement highlights the importance of positive charge for enhancing antibacterial activity, while hydrophobicity is essential for promoting membrane permeation. Further analysis of AMPs against bacteria revealed a high occurrence of lysine (K) residues. Interestingly, AMPs with antibacterial activity against Gram-negative or Gram-positive bacteria showed similar amino acid distributions, suggesting that they may have a similar mechanism of action against different types of bacteria.

**Figure 4:**
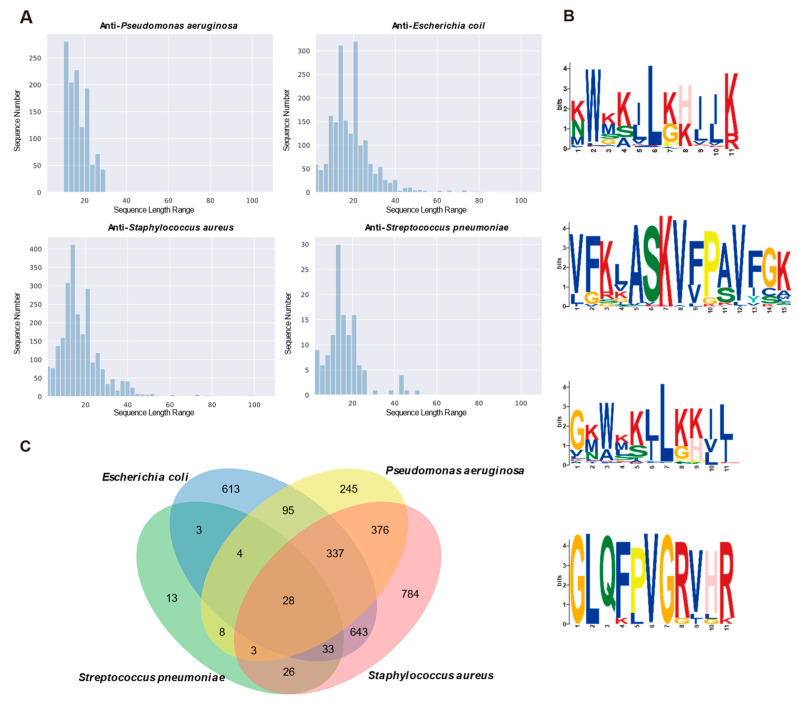
Sequence analysis of AMPs targeting different bacteria strains. (**A**) Percentage distribution of sequence length ranges based on related datasets; (**B**) The amino acid position preference of AMPs targeting various bacterial strains; from top to bottom, they correspond to *P. aeruginosa*, *E. coli*, *S. aureus*, and *S. pneumoniae*; (**C**) A Venn diagram depicting the distribution of AMPs across four bacterial species, namely *P. aeruginosa*, *E. coli*, *S. aureus*, and *S. pneumoniae*.

The lengths of AMP sequences targeting specific bacteria are depicted in Figure 4A, revealing consistency across the four classifications. Analysis of Venn plots (Figure 4C) indicated some overlap among AMPs targeting different bacteria, yet their amino acid abundances varied. Notably, AMPs against *P. aeruginosa*, *E. coli*, and *S. aureus* exhibited a high abundance of lysine and leucine, while those against *S. pneumoniae* showed a predominance of arginine. To delve deeper, we conducted an amino acid position preference analysis using MEME (https://meme-suite.org/meme/tools/meme (accessed on 1 October 2024)). The results (Figure 4B) corroborated previous findings, revealing that arginine was more prevalent in enriched amino acid motifs for *P. aeruginosa*, *E. coli*, and *S. aureus*, whereas it was most abundant in motifs enriched for *S. pneumoniae*. These observations suggested that AMPs exhibit distinct characterization preferences for bacteria with different targets.

### 2.2. Comparison of Different ML Methods on AMP Prediction

To demonstrate the efficacy of our new model, we conducted a comparative analysis against several existing classifiers, including iAMP-CA2L [27], AMPfun [4], iAMP-RAAC [28], dbAMP [12], AMAP [29], and AMPDiscover [30]. All comparisons were conducted using an identical independent test set. These classifiers were primarily constructed using frameworks such as CNN, BiLSTM, SVM, etc., tailored for the recognition of AMPs and their functional classification.

**Figure 5:**
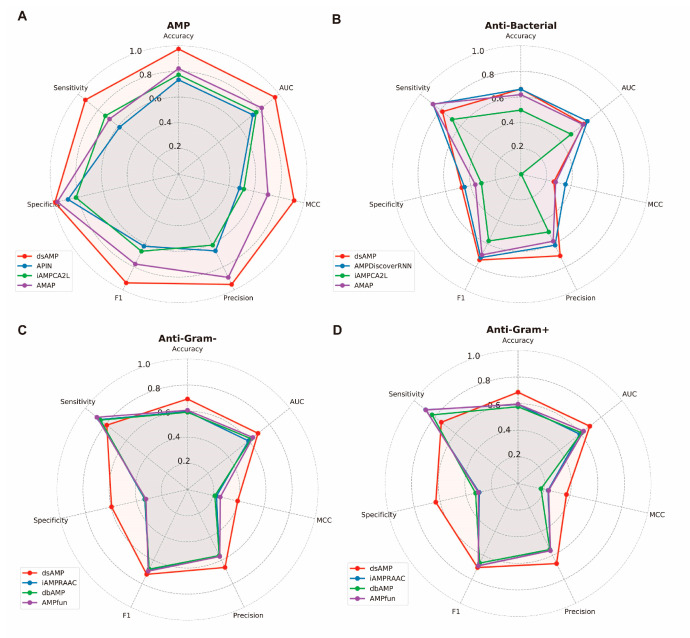
Radar plot for comparing dsAMP with other published models using the independent test set. (**A**) All AMPs datasets; (**B**) Anti-Bacterial AMPs datasets; (**C**) Anti-Gram negative AMPs datasets; (**D**) Anti-Gram positive AMPs datasets.

The experimental results, depicted in Figure 5, highlighted the superior performance of our model, dsAMP, compared to others. Across various metrics, including ACC, MCC, SEN, SPE, PRE, and F1, dsAMP consistently outperformed existing models, indicating its effectiveness in AMP prediction and discovery.

However, in the case of antimicrobial AMP prediction, dsAMP exhibited slightly lower MCC and SEN values. This discrepancy may be attributed to imbalanced data, particularly the scarcity of negative instances [31]. A closer examination of the amino acid distribution revealed similar patterns between positive and negative datasets, posing a challenge for the model to distinguish between them. To address this issue, researchers may employ either oversampling or undersampling techniques. For instance, oversampling techniques (e.g., SMOTE) can achieve balance in a dataset by synthesizing new samples representing the minority class, whereas undersampling methods (e.g., stochastic undersampling) can achieve balance by reducing the number of samples representing the majority class [32,33]. It is anticipated that the implementation of these methodologies will enhance the detection capability of dsAMP with respect to minority class samples, reinforce the robustness of the model, and facilitate an evaluation of its capacity for generalization across disparate datasets. This will provide crucial insights into the performance and reliability of the proposed model in addressing data imbalance.

Nevertheless, dsAMP excelled in AMP classification for both Gram-positive and Gram-negative bacteria, notably achieving the highest specificity. This suggests clear boundaries between different categories in the dataset, facilitating accurate classification. Despite the high-quality data enabling adequate feature capture, the abundance of negative datasets led to a model bias toward predicting dominant negative examples, resulting in lower sensitivity [34].

In summary, dsAMP demonstrates superior performance in predicting AMPs and their functional classification, showcasing its potential as an effective tool in antimicrobial peptide research.

### 2.3. Performance of Transfer Learning AMP Prediction Network Targeting Different Bacteria

**Figure 6:**
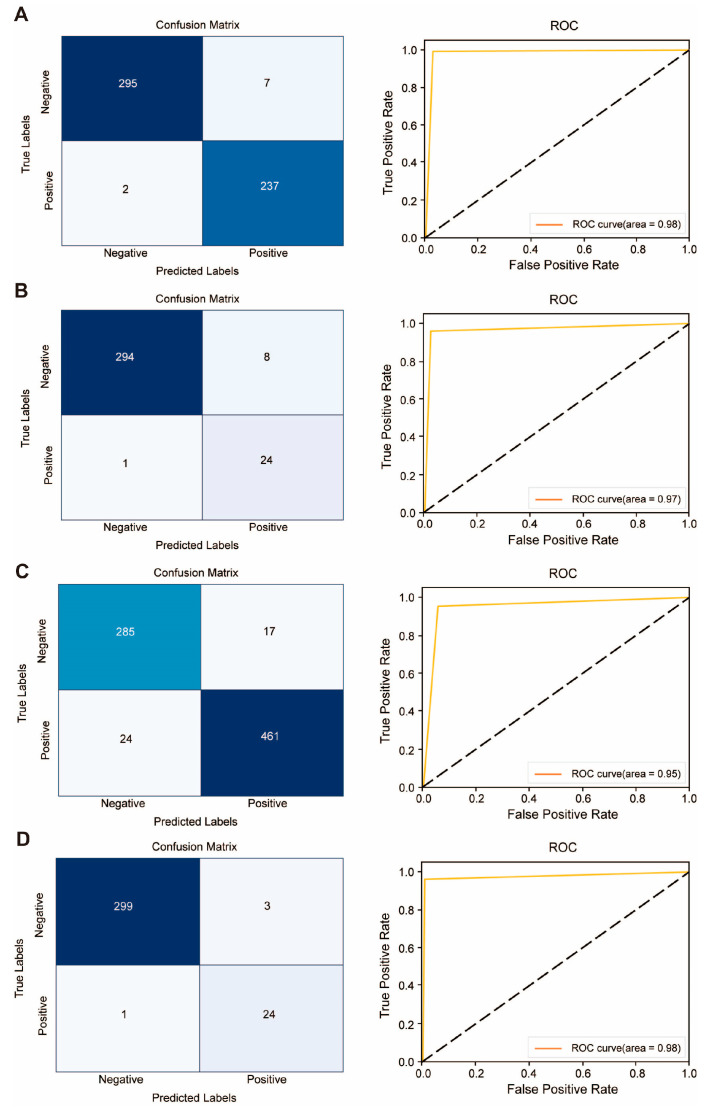
Confusion matrix and ROC curves of AMP classification models targeting different bacte-ria. (**A**) *P. aeruginosa*; (**B**) *E. coli*; (**C**) *S. aureus*; (**D**) *S. pneumoniae*.

AMPs targeting a single bacterium often face challenges due to limited training data and imbalanced datasets, thus making direct training difficult. To overcome this challenge, we fine-tuned the training network using a customized network specifically tailored for AMP identification. Figure 6 depicts the confusion matrix and ROC curves on their respective test sets, while Table 1 provides an overview of the AMP classification networks evaluated for four different bacteria. Overall, all metrics exhibited strong performance, with classification networks targeting *P. aeruginosa* and *S. pneumoniae* demonstrating exceptional accuracy and F1 scores.

Distinctive features in terms of amino acid abundance contributed to the enhanced performance of AMPs targeting these two bacteria. For instance, *P. aeruginosa*-resistant AMPs exhibited significantly higher levels of lysine (K), whereas *S. pneumoniae*-resistant AMPs showed a higher proportion of arginine (R). These unique amino acid profiles enabled the network to acquire more accurate features, resulting in improved classification.

The results underscore the effectiveness of target-specific training using transfer learning techniques, even with a small training dataset. Moreover, the adoption of transfer learning substantially reduces the number of training cycles, thereby saving training time and enhancing the model’s performance during the identification process of AMPs targeting individual bacteria.

### 2.4. Evaluating GAN-Designed Peptides

Despite their different origins, functions, structures, and sequences, the reported AMPs have similar physicochemical properties, mainly possessing three primary traits: positive charge, hydrophobicity, and amphiphilicity. The antimicrobial potency is enhanced with the increasing charge, yet excessive hydrophobicity can induce mammalian cell toxicity. Amphiphilicity is pivotal for peptide structure, impacting both antimicrobial effectiveness and hemolytic potential [3]. We validate the learning effect of the GAN by comparing the generated peptides with a dataset of known AMPs. The data-driven physicochemical properties and performance statistics are used to assess the similarity of the GAN output to the training data.

**Figure 7:**
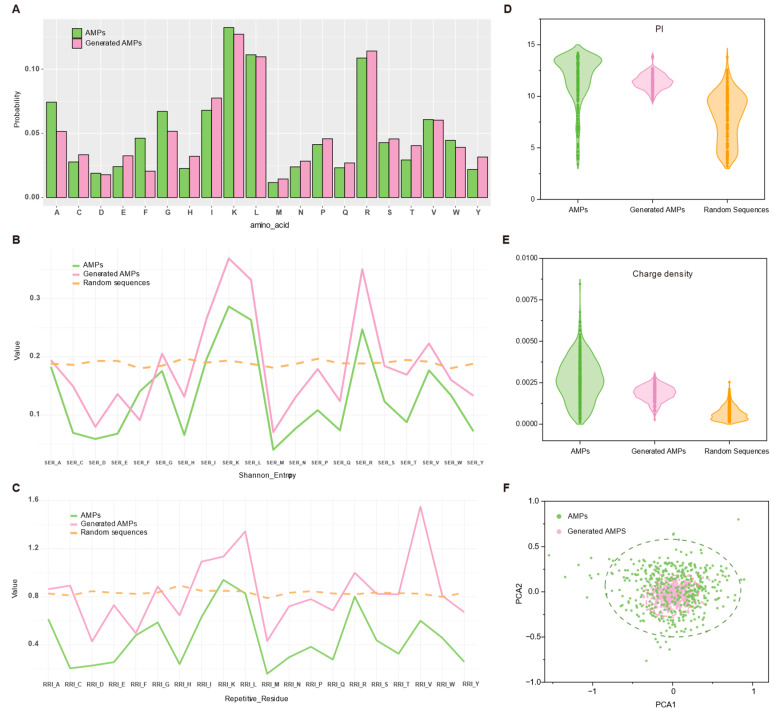
Evaluating GAN-generated peptides in silico. (**A**) Bar plots of the amino acid distribution of generated AMPs and real AMPs; (**B**) Shannon entropy of all the residues of generated AMPs, real AMPs and random peptides; (**C**) Residue repeats patterns for generated AMPs as compared to real AMPs and random peptides; (**D**) Violin plots of PI of generated AMPs, real AMPs, and random peptides; (**E**) Charge densities of PI of generated AMPs, real AMPs, and random peptides; (**F**) Plot of PCA of the physio-chemical properties of generated AMPs and real AMPs.

Figure 7A shows the amino acid distribution of both generated and authentic AMPs, where the abundance of lysine and arginine contributes to positive charge and leucine enhances hydrophobicity. The similarity in amino acid ratios, particularly the enrichment of lysine, arginine, and leucine in both generated and authentic AMPs, indicates a distinctive pattern in the generated sequences akin to authentic AMPs, rather than random generation. Additionally, Figure 7D,E reveal comparisons of isoelectric points (PI) and charge densities, showing that the generated AMPs closely resemble real AMPs, with higher distributions in both parameters compared to random sequences [35]. Furthermore, the secondary structure of peptides, including α-helices, β-sheets, and random coils, is of paramount importance for their biological function and stability. α-helices are particularly renowned for their capacity to penetrate membranes. The uniform hydrogen bonding reliability (uHrel), as evaluated through molecular dynamics simulations, serves as a pivotal indicator of helix stability. Peptides exhibiting elevated uHrel values tend to demonstrate augmented antimicrobial properties [36]. By calculating the uHrel values for different helix groups, the results demonstrated that the relative hydrophobic moments of generated and true antimicrobial peptides (AMPs) were typically greater than 0.2, while those of random peptides were less than this value. This provides further evidence to support the hypothesis that the generated AMPs have similar membrane interactions with the true AMPs.

Shannon entropy at the protein level quantifies the uncertainty or randomness in amino acid distribution within a protein sequence, reflecting the diversity across its positions. Higher Shannon entropy values signify greater variability in the sequence [37]. Comparing the Shannon entropy and residue repeats of generated AMPs with those of real AMPs can offer insights into the importance of repetitive amino acid sequences for their function. The results (Figure 7B,C) demonstrate that the distribution patterns of peptides generated by the GAN closely resemble those of real AMPs. This trend suggests that the GAN model holds promise in generating peptides with comparable physicochemical properties.

When evaluating the performance of antimicrobial peptides (AMPs), different amino acid properties are critical to their function. Aromatic amino acids improve antimicrobial peptide binding to membranes by increasing hydrophobicity, while neutral amino acids improve peptide stability and solubility and reduce charge effects. Aliphatic amino acids enhance hydrophobicity and promote membrane penetration, while acidic amino acids improve affinity by binding to cationic regions of the membrane. Hydrophobic amino acids further enhance membrane penetration and antimicrobial activity. Polar amino acids increase solubility in the aqueous phase, while positively charged amino acids enhance binding to negatively charged membranes. Cyclic amino acids enhance specific binding by increasing structural stability, while basic amino acids enhance membrane binding and penetration. Considering these properties together, the membrane penetration ability, stability, and antimicrobial activity of antimicrobial peptides can be effectively evaluated [17,18]. We therefore used Principal Component Analysis (PCA) to condense the above nine physicochemical features into two dimensions and visualized them using Origin software [33]. Figure 7F shows the feature distribution of the generated AMPs and the real AMPs. The results show that the generated AMPs are uniformly distributed in the mid-range of the real AMPs, indicating that the GAN-designed peptides have a high similarity to most of the real AMPs. This finding further demonstrates the effectiveness of the model.

## 3. Discussion

This study introduces two significant advancements in antimicrobial peptide (AMP) research: the dsAMP classification model and the dsAMPGAN generative network. These innovations address critical challenges in AMP discovery and design and provide several notable findings and implications:

The dsAMP model, employing a CNN-Attention-BiLSTM framework, outperforms existing classifiers across multiple evaluation metrics, including accuracy (ACC), Matthews Correlation Coefficient (MCC), sensitivity (SEN), specificity (SPE), precision (PRE), F1 score, and area under the curve (AUC). This improvement in accuracy is attributed to the integration of diverse AMP databases and the utilization of the ProtTrans BERT-based pre-trained model [25], which significantly enhances feature extraction from AMP sequences. Furthermore, module splicing within the CNN-Attention-BiLSTM framework enhances model depth [10]. Notably, the architecture of dsAMP surpasses networks solely based on CNN, Attention, or BiLSTM frameworks, enabling better discernment of AMP features for classification. Consequently, dsAMP facilitates more precise identification of AMPs, thereby accelerating the discovery of potential antimicrobial agents.

Challenges posed by small datasets necessitate specific methodological strategies to combat issues like overfitting or limited generalization. Moreover, AMPs tailored to specific bacteria offer practical insights, yet their raw datasets are smaller. Transfer learning emerges as a solution, facilitating superior training for classifications with fewer samples in the training dataset and enabling rapid network convergence [38,39]. Notably, AMP prediction results across all four bacteria demonstrated enhanced performance through transfer learning. Additionally, analysis of amino acid abundance composition revealed distinct preferences in bacteria-specific AMPs, serving as valuable references for designing novel AMPs. However, data imbalance can still have an adverse impact on model performance, especially for minority groups. To address this issue, future research should explore strategies such as oversampling and undersampling. By balancing the dataset, we expect that sensitivity and overall accuracy will be improved, thereby enhancing the robustness and reliability of the model.

The dsAMPGAN network, which utilizes a CNN-Attention framework, represents a significant advancement in the generation of novel AMPs. The peptides produced exhibit physicochemical properties akin to those of real AMPs, including amino acid composition, net charge, hydrophobicity, and charge distribution. This capability facilitates the exploration of new peptide designs and the identification of sequences with potentially superior antimicrobial activity. Further enhancement of dsAMPGAN’s utilization of structural information is expected to improve its efficacy in generating highly active AMPs.

The high classification accuracy of the dsAMP model enables efficient screening of AMP sequences, facilitating the rapid identification of potential antimicrobial candidates and potentially reducing the time required for experimental validation. Additionally, the dsAMPGAN network’s ability to generate peptides with specific properties offers a valuable tool for designing new AMPs tailored to particular biological and therapeutic needs.

Future research should focus on refining data balancing techniques and expanding the dataset for dsAMPGAN to enhance its generalization capability. It is important to examine the correlation between oligomerization, electrostatic interactions, and antimicrobial activity in future research [40,41]. Studies have demonstrated that oligomerized peptides often enhance membrane interactions, leading to improved antimicrobial efficacy. For instance, the peptide LL-37 exhibits significantly enhanced antimicrobial activity upon oligomerization due to its increased capacity to disrupt microbial membranes [42]. Additionally, the amphipathic nature of α-helices facilitates varied electrostatic interactions with negatively charged bacterial membranes, which are critical determinants of antimicrobial effectiveness [41]. Incorporating the effects of both oligomerization and electrostatic interactions on the structural dynamics and stability of peptides could facilitate the discovery of novel antimicrobial peptides (AMPs) with enhanced efficacy. Furthermore, environmental factors, including metal complexation and salt effects, have been shown to significantly impact the secondary structure of peptides [43,44]. The presence of metal ions, such as zinc and copper, has been observed to stabilize specific conformations of AMPs, thereby augmenting their biological activity. For example, interactions between the AMP human beta-defensin 2 and zinc ions have been shown to enhance its antimicrobial potency by stabilizing its β-sheet structure [45]. Similarly, variations in ionic strength can alter electrostatic interactions, thereby affecting peptide folding and activity; elevated salt concentrations may shield charged residues, which in turn impacts the peptide’s ability to interact with bacterial membranes [44]. Thus, future identification and design of antimicrobial peptides should emphasize the role of environmental factors.

In summary, the dsAMP and dsAMPGAN models represent significant advancements in AMP research, offering improved classification accuracy and novel peptide generation capabilities. These developments hold considerable promise for accelerating the discovery and development of new antimicrobial agents, thereby addressing the critical issue of antimicrobial resistance.

## 4. Materials and Methods

### 4.1. Data Collection and Preprocessing

The AMPs and their functional types were collected from several databases including DBAASP [46], dbAMP [12], AMPfun [4], iAMP-CA2L [27], and CAMP [47]. Meanwhile, the negative peptide sequences with a length between 10 and 200 residues were downloaded from the UniProt (http://www.uniprot.org) database [48], and all the keywords containing “antibacterial” and related keywords (e.g., “antigrampositive”) entries were removed. The positive and negative data were further cleaned to filter the sequences containing unusual letters (e.g., ‘B’, ‘Z’, ‘U’, ‘J’, ‘O’, ‘X’, ‘n’, or ‘−’) and the CD-Hit program [31] was used to remove the sequences with 90% sequence similarity. In order to construct the test dataset, we trained and evaluated the AMP and functional activity prediction models by randomly selecting 20% of the samples from the positive and negative datasets, and the remaining 80% of the samples served as the training dataset. The independent dataset of iAMPCN [34] was also applied to compare the differences between the different models. For the generative network of AMPs, AMPs of length less than 30 are selected as inputs of GAN. The randomly generated sequences comprised an equal distribution of common amino acids, totaling 500 sequences, each with a length of 30. The specific sample amounts for the above dataset were shown in Table 2.

### 4.2. Protein-Encoding Method

The ProtTrans protein language model [25] is a pre-trained Transformer-based model, trained on millions of protein sequences, which produces sequence embeddings that can be used as the sole input to a downstream model without much optimization, resulting in a model that performs well in areas such as classification. At the same time, the ProtTrans model has no restriction on the length of the protein. We chose ProtT5-XL-UniRef50 to conduct embedding for the classification model. For the generator model, peptides of length L were transformed into shape matrices (6, L) to highlight their physicochemical properties using the PC6 protein coding method [23] based on six physicochemical property values corresponding to each amino acid. The attribute values were scaled from −1 to 1 with respect to the amp-gan model [49], while sequences shorter than 30 residues were filled at the end with zero vectors called ‘X’ to achieve a consistent length of 30.

### 4.3. AMP Predictor Model Construction

**Figure 8:**
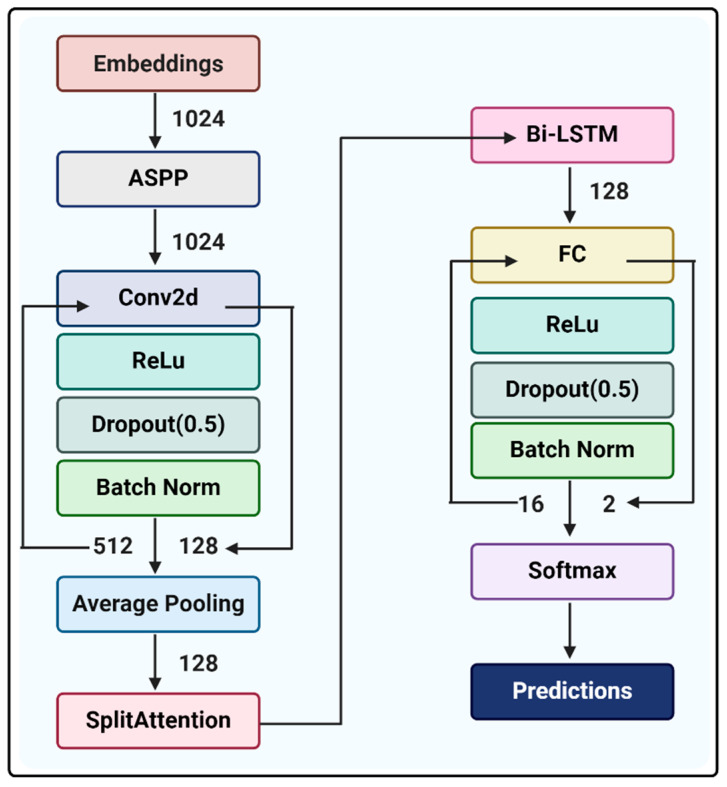
Architecture of dsAMP for identifying AMPs and non-AMPs.

All models were built based on PyTorch. The framework of the model mainly consists of CNN-Attention-BiLSTM (Figure 8). The Atrous Spatial Pyramid Pooling (ASPP) module [50] enhances the receptive field of CNNs by incorporating multiple parallel atrous convolutional layers with different dilation rates. The architecture primarily comprises four convolutional layers and a parallel global average pooling (GAP) layer. It effectively gathers feature maps of varying scales and integrates cross-channel information, thereby expanding the receptive field to facilitate enhanced feature combinations across upper and lower layers. This expanded receptive field enables the integration of more contextual features. Two CNN block architectures are used after ASPP (atrous rates (6, 12, 18)). Each CNN block consists of convolutional layers (kernel size: (3, 3), Padding: (1, 1)) and a rectified linear activation (ReLU) function, dropout layers (rate: (0.5, 0.5)), a batch normalization layer, and finally the SplitAttention [51] module is connected by an average pooling layer. The SplitAttention module is particularly useful in tasks requiring complex feature extraction. It works by dividing the input features into different channels and processing them independently, effectively reducing information loss and facilitating more accurate feature representations, thus significantly improving model performance. Attention is followed by a Bi-LSTM. The two fully connected layers each contain a dropout layer (ratio: (0.5, 0.5)) and a batch normalization layer, and finally the predictions are output via a Softmax function. The batch size of the dataset is set to 256 and training is stopped when the model performance stabilizes. Finally, evaluate the model loss as well as accuracy and save the best model. Hyperparameters are used: loss function (Cross-entropy loss), optimizer (Adam (weight decay = 0.0003)), and learning rate (0.0001).

### 4.4. Transfer Learning for AMP Predictive Modelling

Transfer learning, an ML paradigm, improves the performance of a model on a target task by using knowledge from a related source task [52,53]. In this approach, a model trained on a source task is fine-tuned or adapted to a different but related target task. The model performance, especially in scenarios with limited or costly labeled data for the target task, can be improved by transferring knowledge, such as feature representations or parameter weights. This strategy effectively addresses challenges such as data scarcity and domain adaptation.

Given the limited AMP data for certain bacteria strains (*Pseudomonas aeruginosa*, *Escherichia coli*, *Staphylococcus aureus*, and *Streptococcus pneumoniae*), we employed a transfer learning approach to enhance prediction accuracy in this study. Specifically, we leveraged pre-trained models for antibacterial AMPs and antibacterial AMPs prediction. These pre-trained models served as the foundation, initializing all parameter weights for our bacterial prediction models. The following hyperparameters are used: the batch size of the dataset as 256, optimizer (Adam (weight decay = 0.0005)), epoch (20), and learning rate (0.0001).

### 4.5. AMP GAN Model Construction

**Figure 9:**
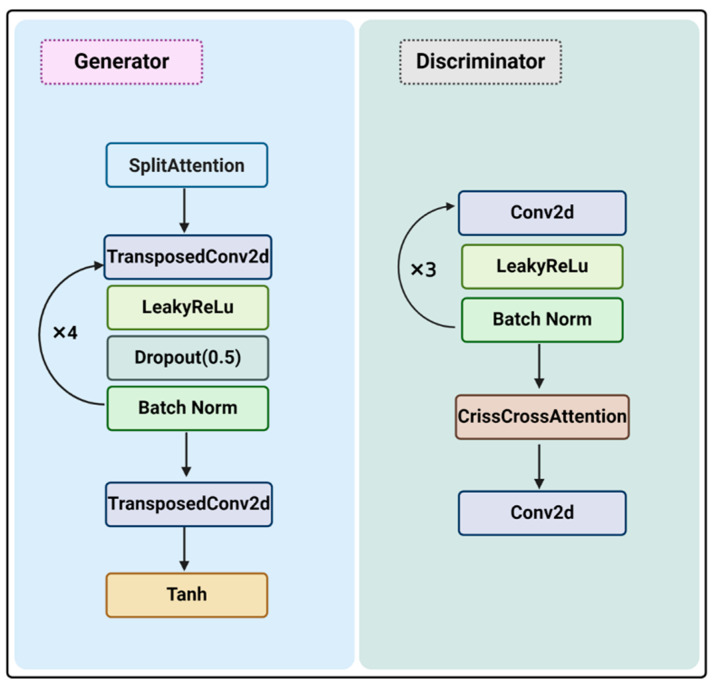
Architecture of dsAMPGAN for generating new AMPs.

GANs, consisting of a generator model and a discriminator model working in parallel, are deep learning-based generative models that use gradient descent to learn the underlying data distribution without prior knowledge of the data structure. The main function of the generator model is to generate samples that mimic the actual data distribution by first generating random samples and then improving them based on feedback from the discriminator module [54]. On the other hand, the discriminator model is used to distinguish between the samples generated by the generator module and the samples in the real data. It takes the real data and the generated data as inputs, determines the authenticity of the data, and feeds it back to the generator [19]. To make the generated output more coherent, improving the accuracy and robustness of the discriminators, the model was modified based on amp_gan [54] by adding an attention mechanism (Figure 9).

The generator consists of a SplitAttention module and five transposed CNN blocks. Each transposed CNN block consists of convolutional layers (kernel size: ((5, 1), (4, 1), (8, 1), (4, 1),(1, 1)), Stride: ((1, 1), (2, 1), (1, 1), (2, 1),(1, 1)), Padding: ((0, 0), (1, 0), (1, 0), (1, 0),(0, 0))) and a rectified linear activation function (LeakyReLU) [55], dropout layers (rate: (0.5, 0.5,0.5,0.5)), and a batch normalization layer. The final transposed CNN block is made up of a 2D transposed convolution layer and a Tanh activation layer.

The discriminator consists of four CNN blocks and a CrissCrossAttention module [31]. Each CNN block consists of convolutional layers (kernel size: ((1, 1), (4, 1), (8, 1), (5, 1)), Stride: ((1, 1), (2, 1), (1, 1), (1, 1)), Padding: ((0, 0), (1, 0), (1, 0), (0, 0))) and a rectified linear activation function (LeakyReLU), dropout layers (ratio: (0.5, 0.5)), and a batch normalization layer. CrissCrossAttention allows different layers of the network to interact with each other in a cross-scale and cross-level manner, thus enhancing the model’s ability to capture local and global contextual information, and also helps the discriminator to capture long-range dependencies and semantic relationships, thus increasing its discriminative power and providing a more accurate and robust evaluation of the generated samples. The final output is conducted by a separate CNN. Hyperparameters are used as follows: the batch size of the dataset is set to 512, optimizer (Adam (weight decay = 0.0005)), learning rate (0.00015).

### 4.6. Model Evaluation

We chose to compute seven metrics to evaluate the classification performance of the predictor: sensitivity (SEN), specificity (SPE), Matthew correlation coefficient (MCC), accuracy (ACC), precision (PRE), F1 score (F1), and area under ROC (AUROC). The seven metrics are defined as shown in Equations (1)–(6):

(1) $$\;\mathrm{Sensitivity}=\frac{\mathrm{TP}}{\mathrm{TP}+\mathrm{FN}}$$

(2) $$\mathrm{Specificity}=\frac{\mathrm{TN}}{\mathrm{TN}+\mathrm{FN}}$$

(3) $$\mathrm{MCC}=\frac{\mathrm{TP}\times \mathrm{TN}-\mathrm{FP}\times \mathrm{FN}}{\sqrt{\left(\mathrm{TP}+\mathrm{FP})(\mathrm{TP}+\mathrm{FN})(\mathrm{TN}+\mathrm{FP})(\mathrm{TN}+\mathrm{FN}\right)}}$$

(4) $$\mathrm{Accuary}=\frac{\mathrm{TP}+\mathrm{TN}}{\mathrm{TP}+\mathrm{TN}+\mathrm{FP}+\mathrm{FN}}$$

(5) $$F1=\frac{2\times \mathrm{Sensitivity}\times \mathrm{Precision}}{\mathrm{Sensitivity}+\mathrm{Precision}}$$

(6) $$\mathrm{Precision}=\frac{\mathrm{TP}}{\mathrm{TP}+\mathrm{FP}}$$

where TP and TN represent the numbers of correctly predicted positive and negative samples, respectively, while FP and FN represent the numbers of incorrectly predicted positive and negative samples, respectively.

For the generative network, physicochemical properties such as amino acid distribution, charge, hydrophilicity distribution, etc. were calculated using the modlAMP package [32] and the Pfeature web server (https://webs.iiitd.edu.in/raghava/pfeature (accessed on 1 October 2024)) [33] to compare the real AMP, randomly generated short peptides, and the generated AMP to evaluate the network. The peptide relative hydrophobic moment μHrel was calculated using the online server http://heliquest.ipmc.cnrs.fr/cgi-bin/ComputParams.py (accessed on 1 October 2024)) [56].

## 5. Conclusions

Our research has contributed significantly to the discovery and development of AMPs in the scientific community. By integrating cutting-edge methods, including the aggregation of state-of-the-art datasets and a sophisticated framework using convolutional neural networks combined with pre-trained natural language models, we have greatly improved the generalizability of our models. As a result, our model becomes a reliable screening tool for identifying novel AMPs in clinical settings, with important implications for drug discovery and therapeutic intervention.

In addition, our studies of AMP generation models have highlighted their critical role in advancing the development of novel antimicrobial drugs. By exploiting the capabilities of these models, we are uncovering new pathways for the synthesis of AMPs with undesirable properties, potentially mitigating the emergence of antibiotic resistance in the clinical laboratory. The combination of dsAMP and dsAMPGAN allows optimization of the entire process from accurate AMP classification to efficient generation. dsAMP can screen for high-potential AMP sequences at an early stage, while dsAMPGAN can generate new variants or optimize existing peptides based on these sequences to improve the overall design and application value of AMPs. In essence, our study lays the foundation for future research efforts and clinical interventions to combat infectious diseases by providing reliable screening tools and facilitating the development of innovative antimicrobial agents.

## Figures and Tables

**Table 1:** Evaluation results of AMPs classification models targeting different bacteria.

Evaluation Indicators	Anti-*Pseudomonas* *aeruginosa*	Anti- *Escherichia* *coil*	Anti-*Staphylococcus* *aureus*	Anti-*Streptococcus pneumoniae*
Accuracy	0.9834	0.9368	0.9479	0.9878
Sensitivity	0.9916	0.9499	0.9505	0.9610
Specificity	0.9768	0.9205	0.9437	0.9901
Precision	0.9713	0.9375	0.9644	0.8889
F1	0.9814	0.9436	0.9574	0.9230
MCC	0.9665	0.8720	0.8905	0.9172

**Table 2:** Summary statistics information of datasets.

Dataset	Negative Samples	Positive Samples
AMP	13,560	63,838
Anti-Bacterial AMP	12,055	10,573
Anti-Gram+ AMP	6607	10,835
Anti-Gram- AMP	6745	10,730

## Data Availability

All our datasets used in this article are uploaded to GitHub website, at https://github.com/zm1999323/dsAMP.git (accessed on 1 October 2024).
